# Targeted Optical Imaging Agents in Cancer: Focus on Clinical Applications

**DOI:** 10.1155/2018/2015237

**Published:** 2018-08-27

**Authors:** Bishnu P. Joshi, Thomas D. Wang

**Affiliations:** ^1^Division of Gastroenterology, Department of Internal Medicine, School of Medicine, University of Michigan, 109 Zina Pitcher Place, BSRB 1722, Ann Arbor, MI 48109, USA; ^2^Department of Biomedical Engineering, University of Michigan, Ann Arbor, MI 48109, USA; ^3^Department of Mechanical Engineering, University of Michigan, Ann Arbor, MI 48109, USA

## Abstract

Molecular imaging is an emerging strategy for in vivo visualization of cancer over time based on biological mechanisms of disease activity. Optical imaging methods offer a number of advantages for real-time cancer detection, particularly in the epithelium of hollow organs and ducts, by using a broad spectral range of light that spans from visible to near-infrared. Targeted ligands are being developed for improved molecular specificity. These platforms include small molecule, peptide, affibody, activatable probes, lectin, and antibody. Fluorescence labeling is used to provide high image contrast. This emerging methodology is clinically useful for early cancer detection by identifying and localizing suspicious lesions that may not otherwise be seen and serves as a guide for tissue biopsy and surgical resection. Visualizing molecular expression patterns may also be useful to determine the best choice of therapy and to monitor efficacy. A number of these imaging agents are overcoming key challenges for clinical translation and are being validated in vivo for a wide range of human cancers.

## 1. Introduction

Cancer is a worldwide health-care concern that is steadily growing. By 2030, an annual incidence and mortality of 21.7 and 13 million cases, respectively, are expected [1]. This increase is attributed to an aging population, greater prevalence of obesity, adoption of western diets by developing countries, and environmental factors [2–4]. Many cancers arise from the epithelium of hollow organs and ducts, including breast, colon, esophagus, head and neck, lung, pancreas, and stomach [5–11]. This thin layer of highly metabolic tissue can be thoroughly and rapidly evaluated in the clinic using methods of optical imaging. Many cancer surveillance guidelines recommend random biopsies, an approach that is inefficient, time consuming, and not widely practiced [12–18]. Targeted optical contrast agents have the potential to provide a molecular mechanism to complement the anatomical view of cancer provided by conventional imaging platforms. They can be administered via different routes, including topically and systemically, to infiltrate the epithelium for effective binding to achieve high contrast images. Malignant and premalignant lesions that may not otherwise be seen can then be detected to guide either diagnostic biopsy or intraoperative surgical resection. Imaging systems should be portable, electrically isolated, and easy to position while providing fluorescence images with micron resolution over a field of view of several centimeters. Progress in this emerging direction requires identification of highly specific targets paired with robust clinical validation.

Molecular imaging is an integrated approach that combines advances in instrumentation with progress in probe chemistries. This methodology promises to advance precision medicine by improving diagnostic performance for early cancer detection, tumor staging, risk stratification, and guidance of therapy. Rapid progress has been made in the technical performance of whole-body imaging systems, including computed tomography (CT), magnetic resonance imaging (MRI), and ultrasound (US) [19–23]. While these platforms provide detailed images of tumor anatomy, they reveal little about the biology that drives cancer progression. Nuclear methods, such as positron emission tomography (PET) and single-photon emission computed tomography (SPECT), visualize and measure physiological processes using radiotracers. For example, 2-deoxy-2-^18^F-fluoro-D-glucose (^18^FDG) is used routinely with PET in clinical practice for cancer staging [24–26]. While both modalities have the capability to image multiple targets using affinity probes labeled with different radioisotopes, this approach is limited by high cost, lack of widespread radiotracer availability, and radiotracer stability. Furthermore, there are limited data to justify use of whole-body PET for cancer screening.

Optical imaging is an alternative modality that detects light emitted from fluorophores attached to ligands that bind specifically to molecular targets overexpressed in cancer. Light is nondestructive, nonionizing, real time, and information rich and can be used over a wide spectral range spanning from visible to near-infrared (NIR). This breadth allows for multiplexing to be performed whereby two or more targets can be visualized simultaneously and is relevant to detection of genetically heterogeneous tumors. Probe platforms are being developed for optical imaging that include small molecule, peptide, affibody, activatable, lectin, and antibody. These ligands range considerably in size from nanoparticles to large macromolecules [27–34]. Tracers used in the clinic for hybrid and theranostic applications have been reviewed previously and are not included in this current review [28]. Chemistries for fluorescence labeling and long-term stability monitoring of these molecules are fairly well developed [22, 35–38]. Clinical translation of these targeted contrast agents is challenging and can be affected by the photophysical properties, stability, pharmacokinetics (PK), and dose. Often, a multidisciplinary team is required [36]. Regulatory expertise is needed to prepare the Investigational New Drug (IND) application. Study objectives for “first-in-human” clinical studies include establishing a safety profile, identifying optimal dosage, determining time course for probe uptake, and validating target expression.

## 2. Nonspecific Optical Imaging Agents

The first optical contrast agents developed for clinical use are nonspecific. Chromoendoscopy employs the use of intravital dyes, such as methylene blue and indigo carmine [39, 40]. These dyes are topically administered and have absorptive properties that highlight mucosal surface patterns. Physician looks for areas with abnormal appearance to guide endoscopic resection of premalignant lesions. This procedure has been recommended by leading medical societies and international experts for use as an adjunct to conventional white light colonoscopy for colorectal cancer (CRC) surveillance in patients with inflammatory bowel disease (IBD), including ulcerative colitis and Crohn's disease [41, 42]. However, the images generated by these dyes are low in contrast, subjective in appearance, difficult to interpret without substantial training, and prone to inter- and intraobserver variability.

By comparison, fluorescence produces high image contrast that can be used for real-time clinical inspection. Fluorophores with a large molar extinction coefficient, safe toxicity profile, low molecular weight (≤1 kD), and minimal nonspecific binding to normal tissues are best suited for this application. Also, low cost, ready availability, and well-developed labeling protocols are desirable. These contrast agents can be used to localize cancer either intra- or extracellularly based on their size and charge distribution. Delivery can be performed either topically or intravenously (iv) depending on the clinical application. Fluorescein isothiocyanate (FITC), a fluorescein derivative that is FDA-approved for human use, is one of the first optical imaging agents used in the clinic [43]. However, the peak absorbance of FITC is near that of hemoglobin, resulting in limited imaging depth and contrast and high autofluorescence background. Also, FITC is sensitive to photobleaching, which limits the time available for imaging.

**Figure 1:**
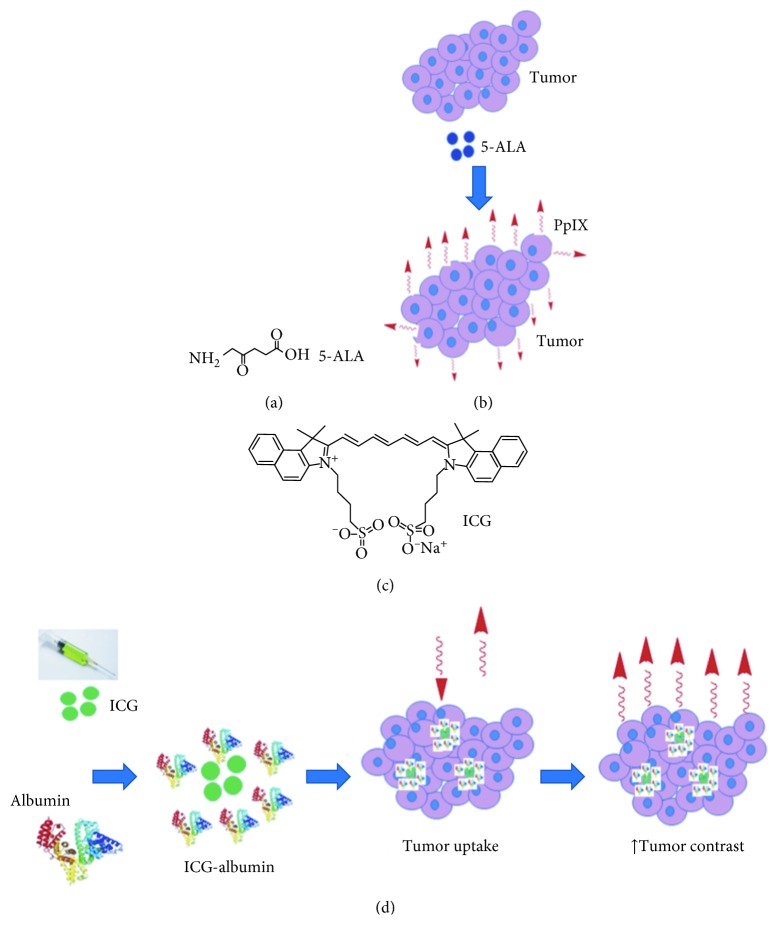
Nonspecific optical imaging agents. (a) Chemical structure of 5-ALA. (b) 5-ALA is taken up by tumor cells and used for synthesis of PpIX (abs = 405 nm, em = 635 nm). (c) Chemical structure of ICG (abs = 783 nm, em = 813 nm). (d) ICG binds to albumin and forms a complex that accumulates in tumor cells to enhance image contrast.

5-Aminolevulinic acid (5-ALA) is an endogenous substrate that emits no fluorescence in its native state. Metabolically active tumor cells preferentially take up 5-ALA for heme synthesis (Figure 1(a)). Protoporphyrin IX (PpIX, *λ*_ex_ = 380 nm; *λ*_em_ = 637 nm) is a downstream substrate that is highly fluorescent (Figure 1(b)). Clinical studies using topical, oral, and intravesical administration have been conducted for a variety of diseases, including glioma, bladder, esophageal, and squamous cell carcinoma. Fluorescence has been collected with a systemic injection of 0.2, 2, and 20 mg/kg in clinical studies of dose escalation. The dose of 20 mg/kg has been found to produce the strongest fluorescence signal from tumor, and margins based on visual and spectroscopic assessment have correlated well with histology. In the clinic, 5-ALA has also been administered orally 6 hours in advance of imaging for detection of bladder cancer and glioblastoma [44–46]. However, studies have shown that 5-ALA and PpIX can accumulate in nonmalignant tissues as well as in tumor, including brain parenchyma, subependymal zone, and choroid plexus [47, 48]. Other studies have shown that use of this nonspecific contrast agent can generate fluorescence in a heterogeneous spatial pattern that may not correlate with the tumor grade [49–53].

Indocyanine green (ICG, *λ*_ex_ = 783 nm; *λ*_em_ = 813 nm) is another nonspecific contrast agent that is FDA-approved for human use (Figure 1(c)). ICG offers several advantages, including low toxicity (LD_50_ of 50–80 mg/kg in animals) and rapid excretion into bile, and is used routinely in the clinic as iv contrast for angiography and evaluation of cardiac and liver functions [54]. ICG produces NIR fluorescence with peak emission near 800 nm. In this spectral regime, sensitivity to hemoglobin absorption, tissue scattering, and tissue autofluorescence is low, and light penetration depth is high. ICG binds rapidly to albumin in circulation and results in 5–10 nm complexes that accumulate in tumors via the enhanced permeability and retention (EPR) effect (Figure 1(d)) [55–57]. ICG has been used clinically to guide surgical resection of cancer, including breast, CRC, and hepatocellular carcinoma [58]. Liberale et al. evaluated the role of fluorescence imaging using an intraoperative injection of free ICG for detection of peritoneal metastases from CRC. Free ICG at 0.25 mg/kg was iv injected, and the mean tumor-to-background ratio (TBR) was 1.92 ± 0.67 in malignant and 1.02 ± 0.06 in benign nodules (*P*=0.0099) in *n*=42 nodules from *n*=9 patients with nonmucinous adenocarcinoma [57]. However, nonspecific dye retention can reduce diagnostic performance for ICG, and clinical utility is limited by high levels of binding to plasma proteins (98%), low stability in aqueous media, and concentration dependent shifts in wavelength [59].

## 3. Targeted Optical Imaging Agents

**Figure 2:**
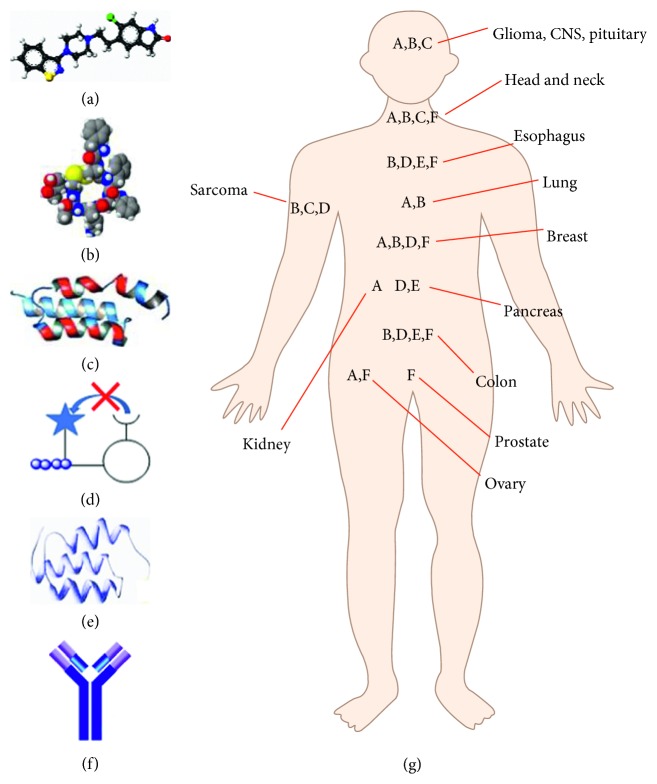
Molecular probe platforms. Targeted contrast agents being developed for optical imaging include (A) small molecule, (B) peptide, (C) affibody, (D) activatable, (E) lectin, and (F) antibody. (G) Clinical studies are being performed using each platform in a wide range of cancers.

By comparison, molecular probes that either bind or are activated by enzymes unique to cancer targets provide specific detection. In general, these contrast agents consist of a ligand, fluorescence signaling moiety, and carrier molecule. Different types of ligands include small molecule, peptide, affibody, activatable, lectin, and antibody (Figure 2(A)–(F)). Clinical studies are being performed to evaluate specific agents in various types of cancer (Figure 2(G)). The probes best suited for clinical imaging have good binding affinity, specific uptake, high cancer retention, and rapid clearance from nontarget tissues. These properties produce high in vivo TBR. In general, molecules with smaller size have a favorable pharmacokinetic profile with faster clearance that allow for imaging to be performed at earlier time points after administration. Long-term stability, in vivo integrity, ease of preparation, and safety are also important features.  Table 1 summarizes clinical trials that use each class of imaging agent and are either ongoing or have been completed and are registered online at Clinicaltrials.gov.

### 3.1. Small Molecule

**Figure 3:**
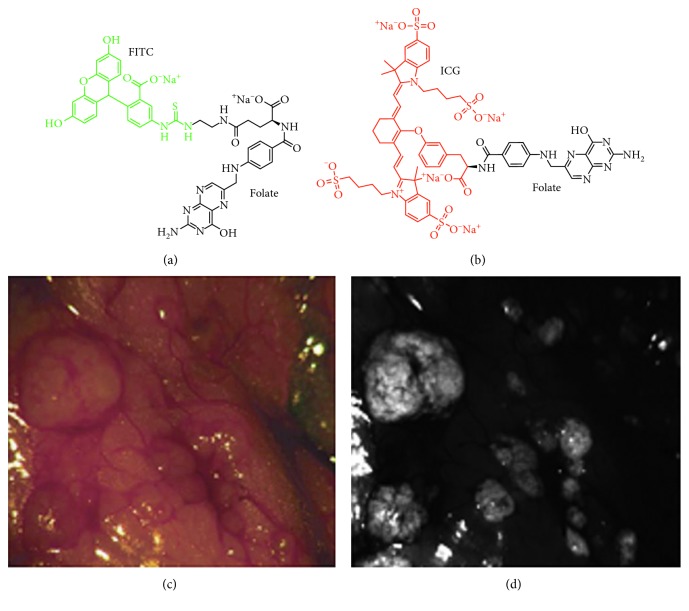
In vivo optical imaging with folate. Chemical structure is shown of folate labeled with (a) FITC (abs = 490 nm, em = 525 nm), known as EC17, and (b) ICG (abs = 783 nm, em = 813 nm), known as OTL38. (c) White light laparoscopic image of peritoneum in vivo and corresponding (d) fluorescence image show enhanced contrast from ovarian cancer metastases following systemic administration of EC17 ((c) and (d) reprinted with the permission from [60]).

Folate is a small molecule that binds specifically to the folate receptor alpha (FR-*α*). This target is overexpressed in ovarian cancer in up to 95% of patients. Moreover, FR-*α* is minimally expressed in normal cells and thus has potential to generate high image contrast. Folate has been labeled with FITC (EC17, *λ*_ex_ = 490 nm; *λ*_em_ = 520 nm) and ICG (OTL38) to target FR-*α* for real-time cancer detection (Figures 3(a) and 3(b)) [60]. In a clinical study of *n*=12 patients with ovarian cancer undergoing exploratory laparotomy, high uptake of EC17 was found in FR-*α* positive tumors and metastases during laparoscopy (Figures 3(c) and 3(d)) [61]. The iv injected formulation for both EC17 and OTL38 appeared to be safe. The fluorescence intensity was found to peak within the range of 2–8 hours postinjection. All patients completed the study, and no serious adverse events (SAEs) were reported. The mean TBR was 3.1 and 4.4 for EC17 and OTL38, respectively, at the injected doses of 0.3 mg/kg for EC17 and 0.05 mg/kg for OTL38. Surgeons were able to find and resect an additional 29% of malignant lesions that were not identified using conventional white light. Based on the preclinical imaging and biodistribution experiments performed with TC1-implanted murine lung cancer cells, the majority of EC17 and OTL38 accumulated in the digestive system, mostly localized in the stomach and small and large intestines. There was significant fluorescence in tumors, and no signal was found in the lung, heart, spleen, muscle, bone, fat, or liver. OTL38 was fluorescent in the kidneys, whereas EC17 was not. There were no signs of acute toxicity in any of the animals. Use of fluorescence did not interfere with the surgeon's ability to perform the procedure. This “first-in-human” study demonstrates potential for use of small molecules to target ovarian cancer, guide radical cytoreductive surgery, and improve methods for intraoperative staging [62–69].

**Figure 4:**
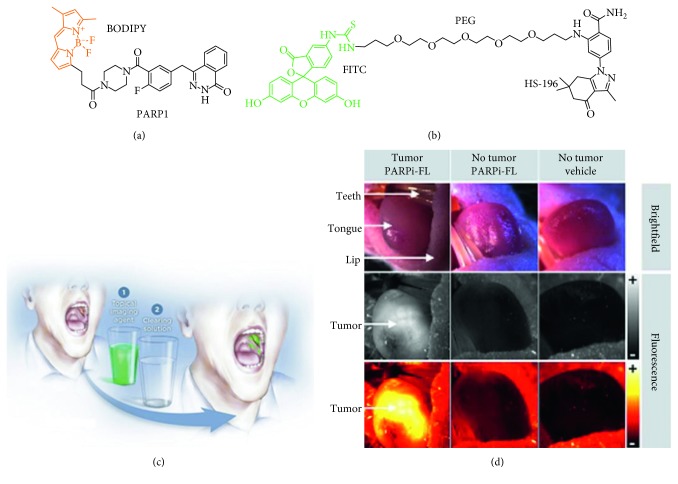
Small molecular inhibitors. Chemical structures of (a) PARP1 labeled with BODIPY (abs = 507 nm and em = 525 nm) and (b) Hsp90 inhibitor HS-196 labeled with FITC. (c) Fluorescence imaging of the oral cavity is performed with topical application of PARPi-FL followed by an acetic acid rinse to remove any unbound contrast agent. (d) Detection of OSCC in mouse tongue in orthotopic xenograft model using fluorescence stereoscope ((c) and (d) reprinted with the permission from [70]).

PARPi-FL is a small-molecule inhibitor that binds to the DNA repair enzyme poly(ADP-ribose) polymerase 1 (PARP1) and is fluorescently labeled with boron-dipyrromethene (BODIPY, *λ*_ex_ = 507 nm; *λ*_em_ = 525 nm) (Figure 4(a)). PARPi-FL has potential to improve diagnostic performance and guide surgical resection of oropharyngeal squamous cell cancer (OSCC) (Figures 4(c) and 4(d)) [70]. The incidence of this disease has increased steadily as a result of chronic infection with the human papillomavirus (HPV). PARP1 expression is increased by ∼8-fold in OSCC relative to normal oral mucosa. In the preclinical study performed in an orthotopic tongue tumor model of OSCC (FaDu cells), iv injection of 75  nmol PARPi-FL per animal provided a maximum uptake at 90 minutes postinjection. Fluorescence imaging showed strong PARPi-FL accumulation in parts of the tongue that were visibly affected by OSCC, whereas no signal accumulation was observed in regions without tumors after injection of either PARPi-FL or vehicle. When compared with vehicle, the average signal intensity from tumor-bearing mice was significantly higher (35.4  ±  8.6  versus 15.2  ±  5.0 AU, resp.; *P* < 0.001). There was no difference between the average signal intensity after PARPi-FL or vehicle injection in tongue and thigh muscle. Based on these promising preclinical results, a Phase 1/2 clinical trial is being performed using PARPi-FL as a targeted contrast agent with topical administration for in vivo imaging (Table 1).

Heat shock protein 90 (Hsp90) is a chaperone that aids in the folding, stabilization, and degradation of cellular proteins and is found in virtually all living organisms. Hsp90 expression is particularly high in cancer cells and may facilitate tumor invasion. HS-196 is a small molecule inhibitor that has been tethered to FITC via a PEG linker for optical imaging to target Hsp90 expressed ectopically in tumors (Figure 4(b)) [71]. Hsp90 has 3 structural domains including an *N*-terminal domain that contains an ATP binding site. Preclinical studies in breast cancer showed that these tethered inhibitors selectively recognize and are internalized by cells that overexpress Hsp90. High uptake of HS-196 was observed in vitro and in vivo in multiple breast cancer cell lines versus the Huh7 liver cancer cell. In vivo imaging of HS-196 with iv injection resulted quick and strong tumor accumulation and long retention. Pharmacokinetic studies show dose dependent uptake of either the visible or NIR forms, peak intensity within the tumor mass by 30 minutes, and a detectable signal for up to 72 hours. The TBR was calculated using flow cytometry, and ∼3-fold greater uptake was observed in isolated tumor cells versus either splenocytes or hepatocytes. A clinical trial using iv administration of HS-196 is planned for breast cancer patients (Table 1).

### 3.2. Peptide

**Figure 5:**
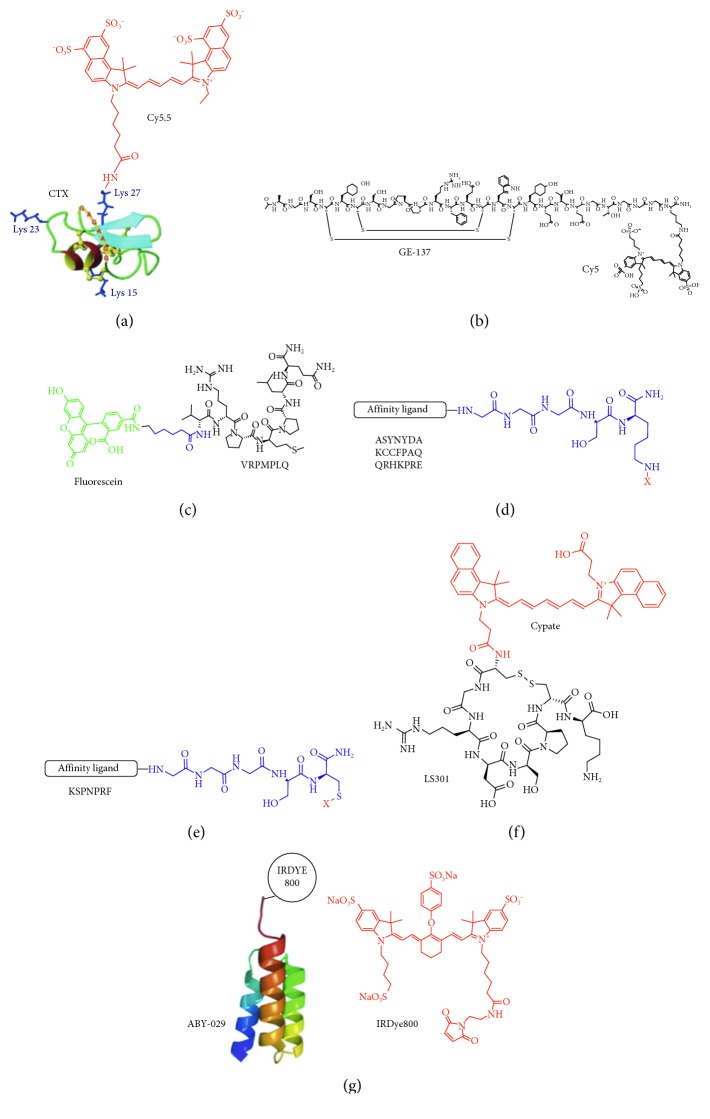
Peptides. (a) Chlorotoxin (CTX) is labeled with Cy5.5 (abs = 675 nm and em = 695 nm), also known as BLZ-100. (b) GE-137 is specific for c-Met and is labeled with Cy5 (abs = 645 nm and em = 665 nm), also known as EMI-137. (c) VRPMPLQ is labeled with fluorescein (abs = 490 nm and em = 520 nm). (d) Peptides ASYNYDA, KCCFPAQ, and QRHKPRE are labeled with either FITC or Cy5 via a linker (X = FITC or CY5). (e) Peptide specific for Her2 is labeled with IRDye800 via a thiol-maleimide (X = IRDYE800). (f) Cyclic peptide LS301 specific for integrin is labeled with cypate (abs = 778 nm and em = 805 nm). (g) ABY-029 affibody specific for EGFR is labeled with IRDye800 ((a) reprinted and modified with the permission from [102] and (b) reprinted with the permission from [78]).

Peptides bind a broad range of cell surface targets with high specificity and affinity. Their relatively small size (<5 kDa) facilitates delivery to overexpressed cancer targets for in vivo detection. Peptides exhibit rapid peak uptake, clear quickly to avoid toxicity, and minimize biodistribution to nontarget tissues. They bind within a few minutes, a timescale that is compatible with clinical use in high volume procedures, such as endoscopy. Peptides have low likelihood to elicit an immune response which allows for repeat use. Peptide analogs with good stability, binding properties, and pharmacokinetic behavior have been used clinically to image neuroendocrine tumors, adenocarcinomas, lymphomas, and melanomas [97–99]. Peptides can be labeled with a variety of fluorophores, including visible and NIR dyes. Recently, cyanine-based dyes that emit fluorescence in the NIR spectrum have been used in the clinic for greater imaging depth [22, 100, 101]. Chlorotoxin (CTX) is a naturally occurring 36-amino acid peptide with 4 disulfide bonds and is derived from the *Leiurus quinquestriatus* scorpion. CTX binds to a lipid raft-anchored complex that contains matrix metalloproteinase-2 (MMP-2), membrane type-I MMP, and a transmembrane inhibitor of metalloproteinase-2 (TIMP2), ClC-3 chloride ion channels, and other proteins [72–74]. This complex is internalized by cancer cells and eliminates functional chloride channels. CTX has been labeled with Cy5.5 (*λ*_ex_ = 675 nm; *λ*_em_ = 695 nm) using a side chain of lysine, denoted as BLZ-100, for use in Phase 1 clinical studies (Figure 5(a)) [75–77, 102, 103]. BLZ-100 is also known as “tumor paint” and was first used to image gliomas. The nonclinical safety and pharmacokinetic profile of BLZ-100 were evaluated in mice, rats, canines, and nonhuman primates (NHP). Single bolus iv administration of BLZ-100 was well tolerated and no-observed-adverse-effect-levels (NOAELs) included 7 mg (28 mg/kg) in rats and 60 mg (20 mg/kg) in NHP. In the most recent study, BLZ-100 was administered at different doses as a single iv bolus 6–31 hours prior to surgery in pediatric glioblastoma patients with either confirmed or suspected brain tumor. Tumor fluorescence was evaluated in situ and ex vivo using a synchronized infrared imaging system (SIRIS). TBR was not reported; however, tumor fluorescence was observed in 13/15 tumors including 5/7 low-grade gliomas. Fluorescence intensity increased with the dose of BLZ-100. More recently, this targeted contrast agent has been used to detect a variety of other cancers, including the medulloblastoma, sarcoma, prostate, colon, breast, lung, and skin.

GE-137 is a 26-mer cyclic peptide, also known as EMI-137, which was developed using phage display screening for specific binding to c-Met (Figure 5(b)) [78]. c-Met is a protooncogene that encodes a transmembrane receptor tyrosine kinase to stimulate tumor progression and metastases. Based on the high prevalence of dysregulation noted in human tumors and its association with advanced disease, c-Met can also be used as a therapeutic target. The peptide was labeled with the NIR fluorophore Cy5 (*λ*_ex_ = 645 nm; *λ*_em_ = 665 nm) and was administered iv for optical imaging of colonic adenomas using a fiber-optic imaging bundle. The biodistribution and pharmacology/toxicity of this peptide were first evaluated in rats and cynomolgus monkeys, and a single iv dose of up to 0.36 mg/kg was felt to be safe in humans. This dose is twice that used for clinical imaging. A single dose (0.02–0.18 mg/kg) of peptide was then administered iv in both healthy volunteers and subjects at high risk of colorectal neoplasia (total *n*=31). The peptide was found to be well tolerated and safe and did not result in any clinically significant changes in symptoms, blood and urinary lab parameters, vital signs, or EKG. Fluorescence measurements indicated a half-life of ∼2 hour 30 minutes for the background to clear at all doses. From ex vivo images, ∼2.3-fold higher fluorescence intensity was observed in the premalignant lesions versus normal colonic mucosa. This imaging approach was demonstrated in vivo in *n*=15 patients and was found to improve the diagnostic yield of adenomas by 19%. Some premalignant lesions had either flat or subtle features on colonoscopy and were easily missed with conventional white light illumination.

Peptides have also been identified empirically using phage display by screening against cancer cells. This approach maximizes fluorescence signal needed for real time in vivo imaging; however knowledge of the target may be incomplete. The peptide VRPMPLQ was labeled with fluorescein via an aminohexanoic acid linker (Figure 5(c)) and was found to bind preferentially to premalignant rather than normal colonic crypts using confocal endomicroscopy [80]. This peptide was applied topically at 100 *µ*M, and imaging was performed after 5 min postincubation. A TBR of 17.9 ± 4.2 (SEM) in fluorescence intensity between adenomas and normal colonocytes was found with an average signal-to-noise ratio (SNR) of 9.3 ± 0.9 (SEM) for *n*=18 adenomas. Contrast ranged between 0.9 ± 17.2 (SD) and 52.3 ± 6.8 (SD).

The peptide ASYNYDA was found to localize to regions of high-grade dysplasia and esophageal adenocarcinoma in patients with Barrett's esophagus using either confocal endomicroscopy or wide-field endoscopy (Figure 5(d)) [82, 83]. The pharmacology/toxicology study was performed in rats at 4 doses in escalation by oral gavage and showed no peptide-related acute adverse effects in clinical signs or chemistries or on necropsy up to 15 days after peptide administration up to 0.86 mg/kg. The receiver-operator characteristic (ROC) curve for in vivo imaging showed an optimum sensitivity of 75% (95% CI: 43%–95%) and specificity of 97% (95% CI: 85%–100%) at TBR = 4.2, with an area under curve (AUC) of 0.91. The performance of the peptide varied with threshold. At this TBR, *n*=9 true positives, *n*=1 false positive, *n*=34 true negatives, and *n*=3 false negatives for identifying neoplasia were found, resulting in a PPV of 90% and NPV of 92%.

**Figure 6:**
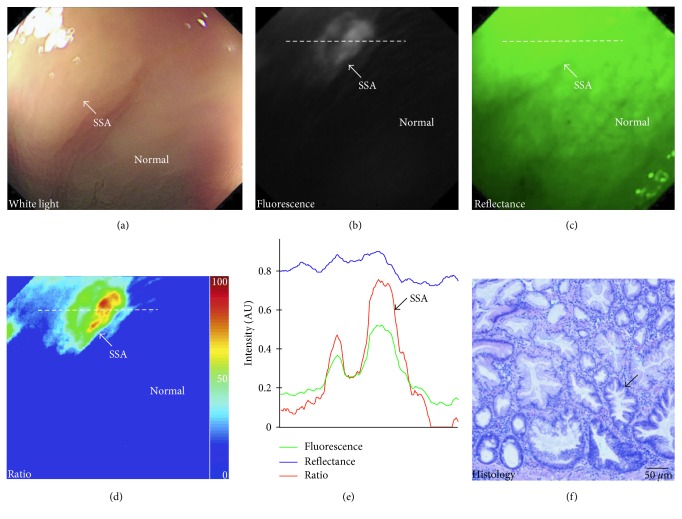
In vivo peptide image of human colonic adenoma. (a) SSA (arrow) with flat morphology collected with conventional white light is shown. (b) Fluorescence image following topical administration of peptide KCCFPAQ labeled with FITC shows increased contrast from lesion (arrow). (c) Reflectance and fluorescence images are combined as a (d) ratio to quantify image. (e) Image intensities along horizontal dashed line in (b–d) show a peak located at site of the SSA (arrow). (f) Corresponding histology of SSA shows serrated morphology (arrow) (reprinted with the permission from [81]).

The peptide KCCFPAQ was screened against human CRC cells that have a point mutation in the V600E locus of the BRAF gene, a characteristic of sessile serrated adenoma (SSA) but not tubular adenomas (Figure 5(d)) [81]. A rigorous pharmacology/toxicology study of GMP-synthesized peptide was performed in rats to provide an initial assessment of safety. Intracolonic administration with a volume of 10 mL/kg was performed in 4 groups of rats at 7 weeks of age, including vehicle (PBS) and at 0.0086 mg/kg, 0.026 mg/kg, and 0.86 mg/kg. No acute peptide-related adverse effects in clinical signs, labs, or necropsy were found in any of the animals. A Phase 1A safety study was performed in *n*=25 patients and showed no significant adverse events with the FITC-labeled peptide administered topically to colonic mucosa (Table 1). An optical imaging study was performed in the proximal colon of *n*=38 patients with suspected adenomas using tandem white light and fluorescence endoscopy, Table 1. SSAs were found to have significantly greater mean fluorescence intensity than normal colon. Fluorescence images were used to distinguish SSAs from normal mucosa with 89% sensitivity and 92% specificity at a TBR of 1.16 (Figures 6(a)–6(f)).

Peptides have also been developed that bind specifically to known cancer targets. QRHKPRE was labeled with Cy5.5 to detect epidermal growth factor receptor (EGFR) overexpressed in epithelial cancers (Figure 5(d) [84]). Use of this peptide for imaging has been demonstrated in several preclinical models of cancer, including colon and liver. Clinical studies are under way for early cancer detection in patients at increased risk for colorectal cancer (Table 1). KSPNPRF was developed to bind specifically to epidermal growth factor receptor 2 (ErbB2), [85] also known as Her2, another member of the tyrosine kinase family. Her2 is overexpressed in many cancers including breast, colon, esophagus, and stomach. This peptide was labeled with IRDye800 (*λ*_ex_ = 775 nm *λ*_em_ = 795 nm) using thiol-maleimide chemistry (Figure 5(e)) and safety with topical administration was established in a Phase 1A clinical study (Table 1). Peptides targeting either EGFR or Her2 did not initiate downstream signaling following ligand-receptor binding to support safety for clinical imaging. Also, both peptides did not show any acute toxicity in rats.

LS301 is cyclic octapeptide (D-Cys-Gly-Arg-Asp-Ser-Pro-Cys)-Lys-OH that binds specifically to integrins expressed by tumor cells but not normal. D-cysteine is incorporated to prevent degradation by endogenous serum proteases. This peptide has been labeled with cypate (*λ*_ex_ = 778 nm *λ*_em_ = 805 nm), a hydrophobic NIR fluorophore, Figure 5(f). Tumor cells take up this targeted contrast agent via receptor-mediated endocytosis, and fluorescence images are visualized using special Cancer vision goggles (CVG). LS301 has shown promising results in vivo in preclinical studies [79].

Fluorescence images collected using LS301 identified all tumors in mice (*n*=10) with a mean TBR of 1.21  ±  0.1 and was used to guide real-time resection. The fluorescence signal was significantly higher in tumors than in surrounding tissue (*P* < 0.05) and correlated well with histology. A clinical study has been planned to evaluate use of this peptide in patients with breast cancer undergoing partial mastectomy and biopsy (Table 1). The study aims to use NIR fluorescence images to evaluate the ability of LS301 to predict the presence of positive cancer margins around the mastectomy site and to locate positive sentinel lymph nodes.

### 3.3. Affibody

Affibodies are nonimmunoglobulin, synthetic proteins (∼7 kDa) that are generally larger in size than peptides but smaller than antibodies. Similar to peptides, affibodies exhibit rapid tumor uptake and fast clearance from normal tissues by comparison with antibodies. They also have good stability and binding affinity. The clinical utility of an affibody specific for Her2 has been demonstrated using nuclear imaging in breast cancer patients with recurring metastases [104]. ABY-029 is an affibody specific for EGFR that has been labeled with IRDye800 (*λ*_ex_ = 775 nm *λ*_em_ = 795 nm) for optical imaging (Figure 5(g)) [86, 87]. A microdose injection of ABY-029 was used to delineate human glioma xenograft tumors in nude rats. Optical imaging with ABY-029 outperformed 5-ALA for detection of orthotopically implanted gliomas [88]. ABY-029 has been synthesized for in vivo characterization and will be evaluated clinically in patients with recurrent glioma, head and neck cancer, and primary sarcoma, Table 1.

### 3.4. Activatable

**Figure 7:**
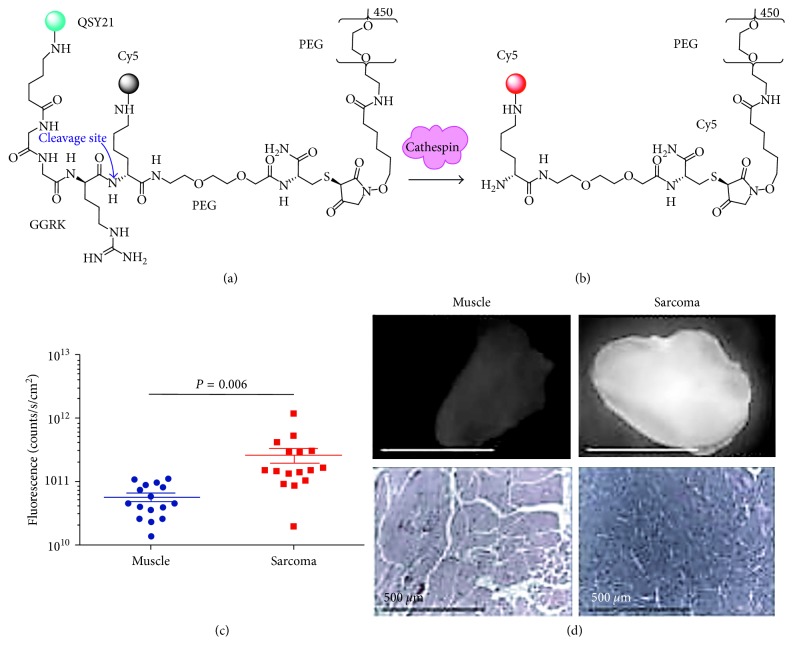
Activatable probe. (a) LUM015 consists of GGRK cleavage site. The Cy5 fluorophore is quenched by QSY21 in native state. (b) Proteolytic cleavage of the quencher by cathepsins activates fluorescence from Cy5. (c) Significantly increased signal is seen in a preclinical model of sarcoma. (d) Representative ex vivo fluorescence images of resected normal human muscle and sarcoma along with corresponding histology ((c) and (d) reprinted with the permission from [89]).

Activatable probes are optically inactive in the native state and become highly fluorescent when cleaved by enzymes that are overexpressed in cancer. This class of targeted contrast agent has been demonstrated in preclinical optical imaging studies in a number of disease models, including cancer, atherosclerosis, rheumatoid arthritis, and thrombosis [34, 105–108]. Lum015 consists of a 22 kDa polyethylene glycol (PEG 450) and the NIR fluorophore Cy5 (*λ*_ex_ = 645 nm *λ*_em_ = 665 nm) attached to a QSY21 (*λ*_ex_ = 660 nm *λ*_em_ = 792 nm) quencher (Figure 7). Cathepsins cleave the Gly-Gly-Arg-Lys (GGRK) recognition sequence to release fluorescence. Preclinical imaging results with LUM015 in various cancer types have been promising. No adverse effects have been found in either mice or canines with naturally occurring cancers following injection 6–24 hours prior to surgery. Biodistribution, pharmacokinetic profiles, and metabolism were similar in mice and human subjects. LUM015 was administered to *n*=6 patients at a dose of 0.5 and 1.0 mg/kg and *n*=3 patients at 1.5 mg/kg. Surgical removal of tumors immediately followed by ex vivo fluorescence imaging was done either the same day as probe injection (at ∼6 hours) or the following day (at ∼30 hours). LUM015 is currently in Phase 1 clinical studies for soft-tissue sarcoma, breast cancer, and digestive tract cancers, including colon, esophagus, and pancreas (Table 1 [89]). Tissue specimens of cancer (*n*=49) imaged intraoperatively showed good correlation with pathology.

### 3.5. Lectin

Lectins are glycoproteins that bind to specific sugar residues (oligosaccharides) and have been developed to detect cell-surface glycans that are altered in cancer [90, 109]. Unlike other posttranslational modifications, glycosylation is highly specific in cancer and provides a promising biomarker for early cancer detection [110, 111]. Wheat germ agglutinin (WGA) is a lectin that has been fluorescein-labeled and topically administered to the epithelial surface of freshly resected specimens of whole human esophagus. Specific binding to high-grade dysplasia was visualized using a clinical fluorescence endoscope ex vivo. These lesions were flat in appearance and not likely to be detected with conventional white light endoscopy. Unlike the other targeting ligands, cancer progression is associated with reduced lectin binding.

**Figure 8:**
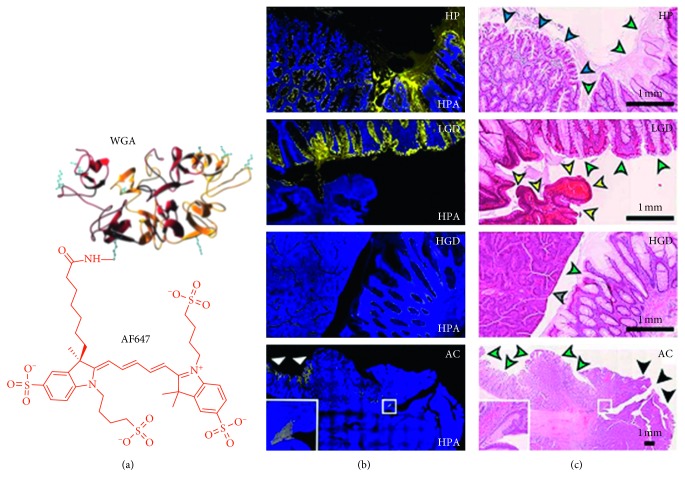
Lectin. (a) Chemical structure is shown of wheat germ agglutinin (WGA) labeled with AF647 (abs = 650 nm and em = 668 nm). (b) Immunofluorescence of human colon, including (HP), low-grade dysplasia (LGD), high grade dysplasia (HGD), and adenocarcinoma (AC), is shown stained with lectin Helix pomatia agglutinin (HPA) labeled with AF647. (c) Corresponding histology (H&E) ((a) reprinted and modified with the permission from [111] and (b) reprinted with the permission from [90]).

WGA labeled with Alexa Fluor 647 (AF647, *λ*_ex_ = 650 nm *λ*_em_ = 668 nm) showed significantly decreased binding in sections of human colonic adenomas ex vivo (Figure 8(a)) [90]. This targeted contrast agent could distinguish among normal epithelium, hyperplastic polyps, low-grade dysplasia, high-grade dysplasia, and adenocarcinoma with high sensitivity, specificity, and positive predictive value (Figures 8(b) and 8(c)). Another lectin, helix pomatia agglutinin (HGA) demonstrated comparable performance. Decreased binding of WGA and HPA to the epithelium in dysplasia suggests that these lectins may be used clinically with fluorescence colonoscopy for early cancer detection. However, this negative contrast strategy can be limited by false-positives when used in vivo because of overlying mucus, anatomic shadows, and mucosal folds. The fluorescein-labeled lectin wisteria floribunda is currently being evaluated in a Phase 1 clinical trial for early detection of colorectal cancer, Table 1.

### 3.6. Antibody

Antibodies are immunoglobulins developed originally for therapy and have been fluorescently labeled for clinical use as targeted imaging agents [33, 91, 112–114]. Following systemic administration, antibodies can have a long in vivo circulation time that can last up to several days. The unbound probe must clear to reduce background before imaging. Cetuximab is a chimeric (human/mouse) monoclonal antibody (mAb), and panitumumab is a fully human mAb specific for EGFR. Both have been labeled with IRDye800 (*λ*_ex_ = 775 nm *λ*_em_ = 795 nm) and are being evaluated clinically (Table 1). Use of cetuximab-IRDye800 to guide intraoperative surgery of head & neck cancer has been demonstrated with NIR fluorescence imaging [92]. This imaging agent was found to be well tolerated and provided high contrast between tumor and normal mucosa in a dose- and time-dependent fashion. Wide-field fluorescence imaging was performed 3-4 days postinjection in *n*=12 patients with squamous cell carcinoma of the head & neck. High-contrast images were collected that could differentiate tumor from normal mucosa during resection. On immunofluorescence, the signal correlated with EGFR expression. In a separate study, an EGFR antibody was labeled with Alexa Fluor 488 (AF488) and topically administered in *n*=40 patients with colorectal neoplasia for imaging with confocal laser endomicroscopy [93]. Targeted biopsies were obtained from each site, and specific binding was found in 94.7% of adenocarcinomas and in 66.7% of adenomas when compared with histology and immunohistochemistry.

**Figure 9:**
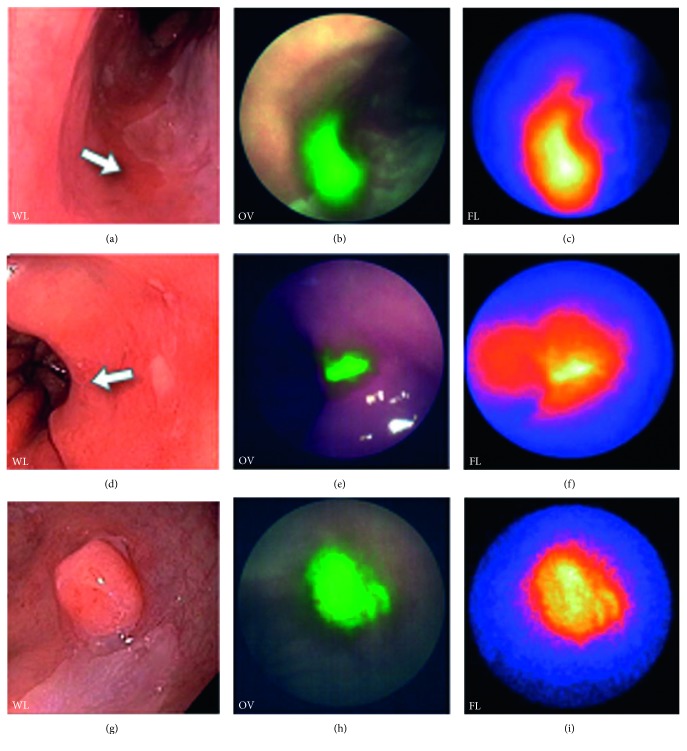
Antibody. Wide-field endoscopic images collected in vivo of human esophageal adenocarcinoma (EAC) following topical administration of bevacizumab labeled with IRDye800. (a) White light (WL), (b) overlay (OV), and (c) fluorescence (FL) image from nonfocal lesion (arrow) is shown. Similar set of images are shown for (d–f) flat and (g–i) protruding EAC (reprinted and modified with the permission from [96]).

Bevacizumab is an mAb that is specific for vascular endothelial growth factor A (VEGF-A) and has been labeled with IRDye800 for in vivo optical imaging to guide surgical resection of breast and pancreatic cancer (Table 1). A Phase 1 clinical study is being performed using this targeted contrast agent to detect premalignant lesions endoscopically in patients with familial adenomatous polyposis (FAP), esophageal adenocarcinoma, and rectal cancer (Table 1). Systemic administration of bevacizumab-IRDye800 has been found to be safe in *n*=20 patients with breast cancer with good uptake at the tumor margin [95]. Fluorescence intensity in primary tumor was found to be higher than that at the tumor margin or in healthy breast. Also, VEGF-A expression with immunohistochemistry was found to correlate with fluorescence intensity. No tumor recurrence was found after surgery guided by bevacizumab-IRDye800 imaging. This molecular probe has also been used to detect premalignant lesions in patients with Barrett's esophagus undergoing endoscopic mucosal resection (EMR), Figures 9–9 [96]. Topical and systemically administered bevacizumab-IRDye800 guided fluorescence endoscopy increased the rate of detection of Barrett's neoplasia by 25%. Many of these lesions were either focal or flat in appearance and were missed by conventional high-definition white-light endoscopy (WLE) and narrowband imaging (NBI). Using iv injection of bevacizumab-800CW, a proof-of-concept study was performed in FAP patients in 3 tracer-dose groups at 4.5, 10, and 25 mg. Patients underwent imaging with fluorescence endoscopy 3 days after injection. The fluorescence intensity was dose-dependent and the 25 mg dose provided a median TBR of 1.84. Bevacizumab-IRDye800CW had a good safety profile, and no tracer-related adverse events were observed.

MDX1201 is a human mAb labeled with AF488 (*λ*_ex_ = 488 nm *λ*_em_ = 520 nm) for specific binding to the extracellular domain of human prostate specific membrane antigen (PSMA). PSMA is a tumor-associated antigen and transmembrane protein that is overexpressed in the membrane of prostate epithelium. This molecular probe is currently being evaluated clinically for image-guided surgery of prostate cancer (Table 1). Girentuximab is an mAb that specifically recognizes carbonic anhydrase IX overexpressed in renal cell carcinoma (Table 1). This dual modality molecular probe can be used for either optical or nuclear imaging [91].

Whole antibodies have been truncated to reduce probe size by forming antibody fragments, diabodies, and minibodies for improved pharmacokinetics [115–119]. Because of their relatively smaller dimensions (<60 kDa), these ligands clear faster from the vasculature to facilitate more rapid visualization of cancer with higher image contrast. Improved serum stability has been achieved with recombinant proteins produced by *E Coli*. After fluorophore conjugation, these ligands maintain the fluorophore closer to the target for improved spatial resolution. Greater effort is being made to develop these antibody-based probes to overcome some of the limitations associated with use of full antibodies for clinical imaging.

## 4. Summary and Outlook

Optical contrast agents that are specific for cancer targets are being developed to visualize molecular behavior in vivo. These fluorescently labeled ligands are being used with optical imaging instruments in the clinic to guide surveillance biopsy and surgical resection. A variety of probe platforms with unique pharmacokinetic properties are being developed to provide specific contrast. Each class of probe offers unique strengths for targeted imaging. Labeling can be achieved over a broad spectrum, and multiplexed detection can be performed to address heterogeneity in target expression. Clinical evaluation of a number of these molecular probes has either been demonstrated or is being planned. Despite the many challenges posed, significant progress has been made toward clinical translation, and continued advances are expected in the near future. Here, we discussed optical imaging agents currently in either clinical trial or development. Many of these agents are being evaluated at an early stage. Phase 2/3 results will determine the likely impact of these agents for the use in general patient population.

Molecularly targeted contrast agents must exhibit properties that include a safe toxicity profile, rapid tumor uptake, higher TBR, and long-term stability to be clinically useful. Complete tumor resection is essential for curative treatment, and evaluation of tumor margins can be challenging. TBR may differ in various clinical studies depending on tumor size, dose, and image acquisition time. The minimal TBR should be achieved to accurately discriminate the lesion from healthy tissues and avoid false positives. Methods to measure TBR should be standardized, and multicenter clinical studies are needed to validate diagnostic performance. Several molecular probes have already overcome key regulatory hurdles and have been FDA-approved for use in first-in-human clinical studies. Preoperative biopsies may be needed to confirm expression of the intended target prior to intraoperative use. Rigorous confirmation of specific probe interaction with the target is imperative. Also, the cost associated with use of exogenous agents, and potential for reimbursement must be considered before widespread acceptance can be expected. Imaging instruments that are sensitive to the spectral response of the fluorophore must become commercially available and easy to obtain by the community physician.

Despite the opportunity for molecular contrast, optical imaging techniques can be limited by tissue penetration depth. Photoacoustic imaging (PAI) is an emerging optical method that is being developed to improve this performance parameter [120–126]. This technology is also sensitive to targeted molecular contrast agents and can be useful for staging of early cancers (T1_a_ versus T1_b_) to guide the choice between local endoscopic versus conventional surgical resection. Preclinical images collected in small animals in vivo for a variety of diseases using small-molecule dyes, gold and carbon nanostructures, and liposome encapsulations have also shown promise with PAI. However, considerable challenges such as the large size of nanostructures relative to physiological barriers, biological requirements, target-tissue retention, and safety profiles must be addressed prior to clinical application.

## Figures and Tables

**Table 1:** Clinical studies of targeted imaging agents. A summary of the ongoing or completed clinical trials, as described online at Clinicaltrials.gov organized by each class of molecular probe.

NCT#	Dates	Cancer	Ligand/target	Fluorophore	Reference
*Small molecule*					
NCT02000778	11/2013–2/2018	Ovary	EC17/Folate-α	FITC	[60, 61]
NCT02769533	09/2015–9/2020	Pituitary	OTL38/Folate-α	ICG	[62]
NCT01778933	05/2013–2/2018	Renal cell	EC17/Folate-α	FITC	[63]
NCT01778920	04/2012–5/2016	Lung	EC17/Folate-α	FITC	[64]
NCT02602119	05/2015–8/2017	Lung	OTL38/Folate-α	ICG	[65]
NCT01994369	05/2014–2/2018	Breast	EC17/Folate-α	FITC	[66]
NCT02653612	01/2016–2/2020	Lung	EC17/Folate-α	FITC	[67]
NCT02645409	12/2015–4/2018	Renal cell	OTL38/Folate-α	ICG	[68, 69]
NCT03085147	03/2015/-3/2019	Head & neck	Olaparib/poly(ADP-ribose) polymerase 1	BODIPY	[70]
NCT03333031	01/2018–2/2020	Breast	HS-196/Hsp90	FITC	[71]

*Peptide*					
NCT02462629	06/2015–2/2016	CNS	BLZ-100/a^*∗*^	Cy5.5	[72, 73]
NCT02496065	07/2015–2/2016	Breast	BLZ-100/a^*∗*^	Cy5.5	[74]
NCT02464332	09/2015–5/2016	Sarcoma	BLZ-100/a^*∗*^	Cy5.5	[75–77]
NCT02234297	10/2014–2/2016	Glioma	BLZ-100/a^*∗*^	Cy5.5	[72, 73]
NCT02097875	12/2013–3/2015	Basal/squamous cell	BLZ-100/a^*∗*^	Cy5.5	[75–77]
NCT03205501	02/2017–2/2018	Esophagus	EMI-137/c-Met	Cy5	[78]
NCT03360461	12/2017–7/2018	Colon	EMI-137/c-Met	Cy5	[78]
NCT02676050	02/2018–6/2018	Lung	EMI-137, NAP/c-Met	Cy5	[78]
NCT02807597	12/2017–2/2020	Breast	LS301/αVβIII integrins	Cypate	[79]
NCT01722058	02/2013–8/2013	Colon	VRPMPLQ/b^*∗*^	Fluorescein	[80]
NCT02156557	06/2014–7/2016	Colon	KCCFPAQ/c^*∗*^	FITC	[81]
NCT01391208	02/2011–6/2012	Esophagus	ASYNYDA/d^*∗*^	FITC	[82]
NCT01630798	07/2012–9/2013	Esophagus	ASYNYDA/d^*∗*^	FITC	[83]
NCT02574858	11/2015–8/2016	Esophagus	QRHKPRE/EGFR	Cy5	[84]
NCT03161418	06/2017–9/2017	Esophagus	KSPNPRF/Her2	IRDye800	[85]
NCT03148119	03/2017–3/2018	Colon	QRHKPRE/EGFR	Cy5	[84]

*Affibody*					
NCT02901925	12/2016–3/2018	Glioma	ABY-029/EGFR	IRDye800	[86–88]
NCT03282461	10/2017–2/2018	Head & neck	ABY-029/EGFR	IRDye800	[86–88]
NCT03154411	08/2017–2/2018	Sarcoma	ABY-029/EGFR	IRDye800	[86–88]

*Activatable*					
NCT02438358	06/2015–9/2016	Breast	LUM015/Enzymes	Cy5	[89]
NCT01626066	06/2012–8/2015	Sarcoma	LUM015/Enzymes	Cy5	[89]
NCT02584244	05/2016–3/2018	Colon, pancreas, esophagus	LUM015/Enzymes	Cy5	[89]

*Lectin*					
NCT03070613	04/2017–3/2018	Colon	Wisteria floribunda/e^*∗*^	Fluorescein	[90]

*Antibody*					
NCT02497599	06/2015/-8/2018	Renal cell	Girentuximab/carbo-nic anhydrase IX	IRDye800	[91]
NCT03134846	05/2017–1/2021	Head & neck	Cetuximab/EGFR	IRDye800	[92]
NCT02736578	07/2016/-4/2017	Pancreas	Cetuximab/EGFR	IRDye800	[92]
NCT01987375	11/2015–2/2018	Head & neck	Cetuximab/EGFR	IRDye800	[92]
NCT02415881	04/2015–8/2017	Head & neck	Panitumumab/EGFR	IRDye800	[92]
NCT01372189	01/2011–7/2011	Colon	anti-EGFR mAb	AF488	[93]
NCT02048150	03/2015–9/2016	Prostate	MDX1201/PSMA	AF488	[94]
NCT02743975	09/2016–2/2017	Pancreas	Bevacizumab/VEGF-A	IRDye800	[95, 96]
NCT01972373	10/2013–1/2017	Rectum	Bevacizumab/VEGF-A	IRDye800	[95, 96]
NCT02583568	10/2015/-2/2017	Breast	Bevacizumab/VEGF-A	IRDye800	[95, 96]
NCT02129933	04/2014–1/2016	Esophagus	Bevacizumab/VEGF-A	IRDye800	[95, 96]
NCT02113202	03/2014–0/2015	Colon	Bevacizumab/VEGF-A	IRDye800	[95, 96]

a^*∗*^: multiple targets reported including matrix metalloproteinase-2 (MMP-2), membrane type-I MMP, and a transmembrane inhibitor of metalloproteinase-2 (TIMP2), ClC-3 chloride ion channels, and other proteins; b^*∗*^–d^*∗*^: peptide was screened using unbiased selection and target is unknown; e^*∗*^: disaccharides and other glycans.

## Abbreviations

5-ALA: 5-aminolevulinic acid
BODIPY: Boron dipyrromethene
CRC: Colorectal cancer
CT: Computed tomography
CTX: Chlorotoxin
CVG: Cancer vision goggles
EGFR: Epidermal growth factor receptor
FAP: Familial adenomatous polyposis
FITC: Fluorescein isothiocyanate
FDG: Fluoro-deoxy-glucose
FDA: Food and drug administration
Her2: Human epidermal growth factor receptor 2
HPA: Helix pomatia agglutinin
Hsp90: Heat shock protein 90
IND: Investigational new drug
IBD: Inflammatory bowel disease
mAb: Monoclonal antibody
iv: Intravenous
MRI: Magnetic resonance imaging
NBI: Narrowband imaging
NHP: Nonhuman primates
NIR: Near-infrared
NOAEL: No-observed-adverse-effect-levels
NPV: Negative predictive value
OSCC: Oral squamous cell carcinoma
PAI: Photoacoustic imaging
PET: Positron emission tomography
PK: Pharmacokinetic
PPIX: Protoporphyrin IX
PPV: Positive predictive value
PSMA: Prostate specific membrane antigen
SD: Standard deviation
SEM: Standard error of the mean
SNR: Signal-to-noise ratio
SPECT: Single-photon emission computed tomography
TBR: Tumor-to-background ratio
US: Ultrasound
VEGF: Vascular endothelial growth factor
WLE: White light endoscopy
WGA: Wheat germ agglutinin.
